# Optical coherence tomography versus intravascular ultrasound in patients with myocardial infarction: a diagnostic performance study of pre-percutaneous coronary interventions

**DOI:** 10.1590/1414-431X20209776

**Published:** 2020-08-17

**Authors:** Zongbao Niu, Xiaolan Lv, Jianhua Zhang, Tianping Bao

**Affiliations:** 1 Color Ultrasonic Room, Affiliated Hospital of Hebei University, Baoding, Hebei, China; 2 Department of Cardiology, Handan Shengji Tumor Hospital, Handan, Hebei, China; 3 Color Ultrasonic Room, the First Central Hospital of Baoding, Baoding, Hebei, China

**Keywords:** Frequency domain optical coherence tomography, Intravascular ultrasound, Myocardial infarction, Percutaneous coronary intervention, Quantitative coronary angiography

## Abstract

Accurate coronary measurements are important in guiding percutaneous coronary intervention. Intravascular ultrasound is a widely accepted diagnostic modality for coronary measurement before percutaneous coronary intervention. The spatial resolution of optical coherence tomography is 10 times larger than that of intravascular ultrasound. The objective of the study was to compare quantitative and qualitative parameters of frequency domain optical coherence tomography (FDOCT) with those of intravascular ultrasound and coronary angiography in patients with acute myocardial infarction. Diagnostic parameters of coronary angiography, intravascular ultrasound, and FDOCT of 250 patients with coronary artery disease who required admission diagnosis were included in the analyses. Minimum lumen diameter detected by FDOCT was larger than that detected by quantitative coronary angiography (2.11±0.1 *vs* 1.89±0.09 mm, P<0.0001, q=34.67) but smaller than that detected by intravascular ultrasound (2.11±0.1 *vs* 2.19±0.11 mm, P<0.0001, q=12.61). Minimum lumen area detected by FDOCT was smaller than that detected by intravascular ultrasound (3.41±0.01 *vs* 3.69±0.01 mm^2^, P<0.0001). FDOCT detected higher numbers of thrombus, tissue protrusion, dissection, and incomplete stent apposition than those detected by intravascular ultrasound (P<0.0001 for all). More accurate and sensitive results of the coronary lumen can be detected by FDOCT than coronary angiography and intravascular ultrasound (level of evidence: III).

## Introduction

Atherosclerosis is the leading cause of myocardial infarction (1), morbidity, and mortality (2) in the Chinese population. It also increases the cost of diagnosis and treatment of patients (3).

Accurate coronary measurements are important in guiding percutaneous coronary intervention (4). Intravascular ultrasound is a widely accepted diagnostic modality in cases of myocardial infarction because it provides moving images, has no risk of radiation dose, is economical, detects atherosclerosis, and quantifies plaque geometry and structure (5) but it is an invasive method and requires experienced cardiologists for interpretation of images (6). Therefore, it is used in a low proportion of percutaneous coronary interventions where gross analysis is possible (6). Quantitative coronary angiography is the standard method for coronary measurement (4). Optical coherence tomography is based on near-infrared interferometry and is a high-resolution intracoronary imaging diagnostic modality (1). The spatial resolution of optical coherence tomography (10–20 µm) is 10 times larger than that of intravascular ultrasound (4). Frequency-domain optical coherence tomography (FDOCT) provides 100 frames/s for imaging of long vessels, which is feasible for diagnosis of coronary plaque (7) but the accuracy and sensitivity of FDOCT are not completely clear (4).

The objective of this analysis was to compare quantitative and qualitative diagnostic data of FDOCT with intravascular ultrasound and coronary angiography for coronary measurement before percutaneous coronary intervention in patients with acute myocardial infarction.

## Material and Methods

### Ethics approval and consent to participate

The designed protocol (FHB/CL/27/19 dated 23 September, 2019) of this study was approved by the First Central Hospital of Baoding Review Board and the Medical Council of China. The study adhered to the law of China and the Strengthening the Reporting of Observational Studies in Epidemiology (STROBE) statement. As a retrospective study, registration in the Chinese clinical trial registry was waived by a local institutional review board. An informed consent form was signed by all participants regarding the diagnosis and publication of the study including personal images and data irrespective of time and language during hospitalization.

### Study population

**Figure 1:**
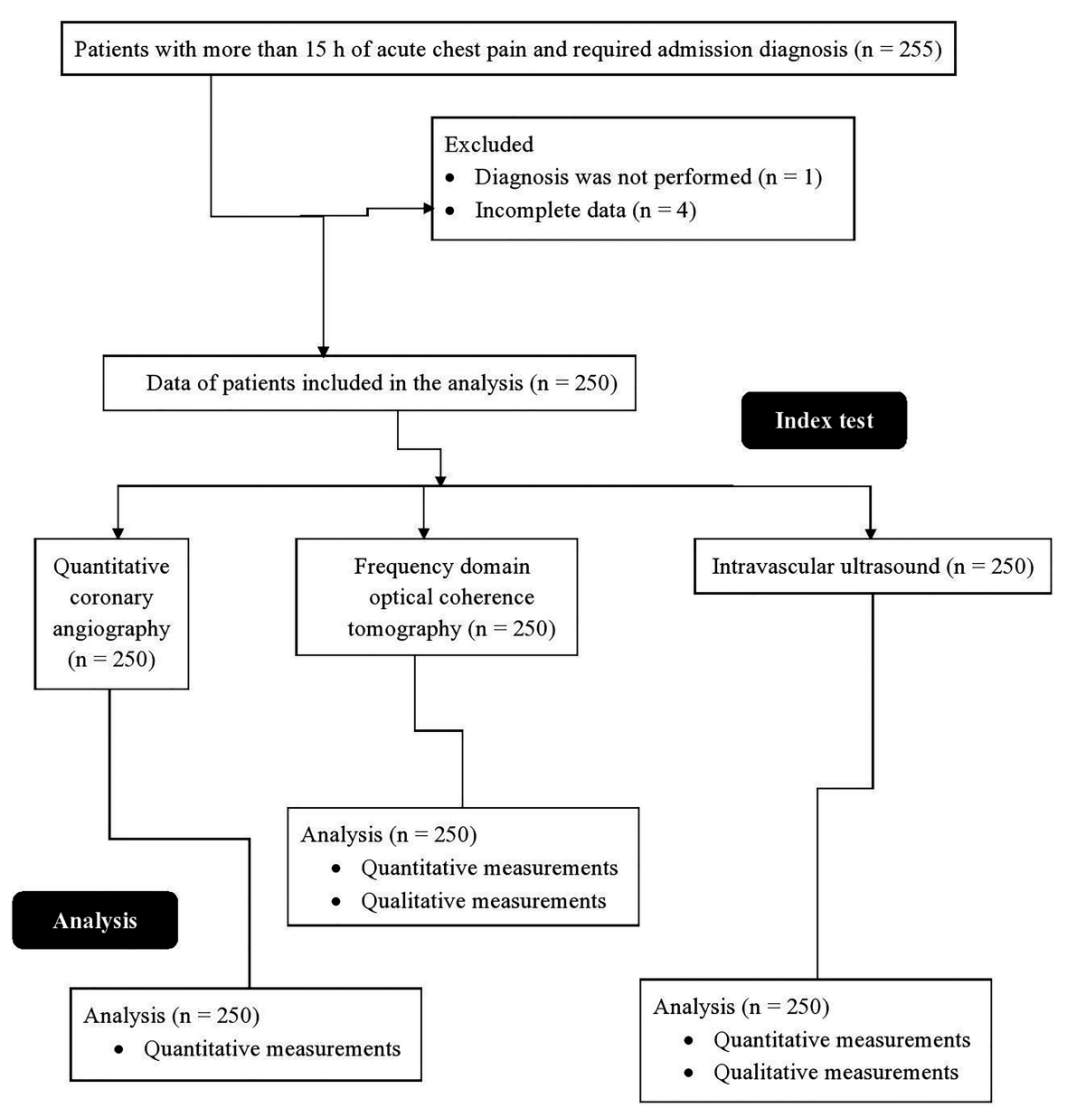
Flow diagram of the study.

From January 15, 2018 to September 1, 2019, a total of 255 patients with more than 15 h of acute chest pain who required admission diagnosis were admitted at the Emergency Department of the Affiliated Hospital of Hebei University (Baoding, Hebei, China), the Handan Shengji Tumor Hospital (Handan, Hebei, China), and the First Central Hospital of Baoding (Baoding, Hebei, China). Complete data of four patients were not available at the Institutes. In one patient, diagnostic catheters were not passed through target lesions. Therefore, the data regarding quantitative coronary angiography, intravascular ultrasound, and FDOCT of these patients were not included in the analysis. Data of 250 patients with acute myocardial infarction (chest pain or discomfort that traveled to the arm, shoulder, back, neck, and or jaws) were included in the analyses (Figure 1).

### Quantitative coronary angiography

With the aid of Infinity^®^ 6F guiding catheters (Cordis Corp., USA), angiography was performed by six-to-eight projections of the left coronary arteries and two-to-three projections of the right coronary arteries. This analysis and the following ones were performed by experienced specialists with at least 3 years of experience.

### FDOCT

C7-XR OCT system (LightLab Imaging, USA) and Cordis Infinity^®^ 6F guiding catheters were used for tomography. A catheter was introduced into a 0.36-mm guidewire (Boston Scientific Corporation, USA). Contrast media was flushed at 4 mL/s for 4 s by an injector pump (4).

### Intravascular ultrasound

Cordis Infinity^®^ 6F guiding catheters were used for ultrasound images. A 40-MHz transducer and a scanner (Philips Healthcare System, USA) were used for the intravascular ultrasound.

### Image analyses

Images were analyzed as per Table 1 (4) by radiologists in consultation with the interventional cardiologists and sonographic technologists of the Institutes. A difference of opinions between observers was solved by a consensus.

**Table 1:** Parameters of image analyses.

Parameters	Quantitative coronary angiography	Intravascular ultrasound	Frequency domain optical coherence tomography
Minimum lumen diameter	Average lumen diameter of the two orthogonal projections without foreshortening	Mean diameter at the minimum lumen area	Mean diameter at the minimum lumen area
Minimum lumen area	Average lumen area of the two orthogonal projections without foreshortening	Smallest lumen area in the selected frame	Smallest lumen area in the selected frame
Intra-stent tissue protrusion	N/A	Prolapse of tissue connecting adjacent struts between stent struts extending inside a circular arc	Prolapse of tissue connecting adjacent struts between stent struts extending inside a circular arc
Incomplete stent apposition	N/A	Clear separation between the vessel wall and at least one stent strut	Distance between the vessel wall (>20 mm of the actual stent thickness) and the center reflection of the strut
Stent edge dissection	N/A	Disruption of the surface of the luminal vessel at the edge segments	Disruption of the surface of the luminal vessel at the edge segments
Thrombus	N/A	Irregular low echoic attached or detached mass	Exit mass with significant attenuation beyond the stent struts into the lumen

N/A: Not applicable.

### Percutaneous coronary intervention procedure-related complications

Data regarding percutaneous coronary intervention procedure-related complications were collected and analyzed. The abrupt closure in the targeted coronary artery was considered as acute coronary occlusion. Embolism due to one or more air bubbles was considered as an air embolism. Blood flow found slow, which was reported normal at the time of diagnosis in the targeted coronary artery, was considered as slow flow. If the contrast agent was found outside the coronary lumen, it was considered as coronary dissection. If haze was found in projections, it was considered as thrombus formation. Sudden vessel occlusion was considered as vasospasm. The abnormal rhythm of the heart was considered arrhythmia (8).

### Statistical analyses

InStat 3.01 GraphPad (USA) was used for statistical analyses. ANOVA was performed to compare numerical variables. Tukey’s test (considering a critical value [q] >3.314 as significant) was used for *post hoc* analysis. The chi-squared independent test was performed for categorical variables. Inter- and intra-rater agreement was evaluated by weighted *k* values (0< k ≤0.2: slight; 0.21≤ k ≤0.4: fair; 0.41≤ k ≤0.6: moderate; 0.61*≤* k *≤*0.8: substantial; and k ≥0.81: perfect) (9). The results of the study were considered significant at a 95% confidence level.

## Results

### Demographical and clinical conditions of patients

A total of 192 (77%) enrolled patients were male and 58 (23%) were female. The mean age of patients was 57.42±9.45 years. The other demographical and clinical parameters are shown in Table 2.

**Table 2:** Demographical and clinical conditions of the enrolled patients.

Parameters	Population
Patients	250
Age (years)	57.42±9.45
Minimum	51
Maximum	70
Gender	
Male	192 (77)
Female	58 (23)
Coronary risk factor	
Diabetes mellitus	143 (57)
Hypertension	121 (48)
Dyslipidemia	101 (40)
Current smoking habit	95 (38)
Family history of ischemic heart disease	41 (16)
Ethnicity	
Han Chinese	230 (92)
Mongolian	16 (6)
Tibetan	4 (2)

Categorical data are reported as frequency (percentage) and numerical data as means±SD.

### Quantitative measurements

**Figure 2:**
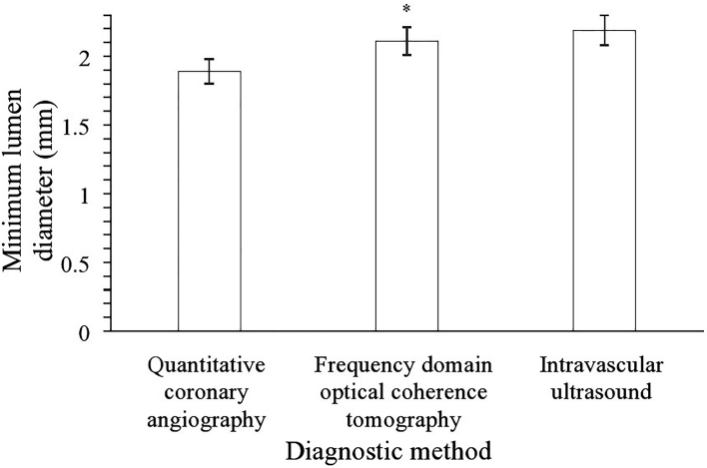
Minimum lumen diameter statistics. Data are reported as means±SD. Data of 250 patients were included in the analysis. *P<0.05 compared to the other diagnostic methods (ANOVA and Tukey *post hoc* test).

Minimum lumen diameter detected by FDOCT was larger than that detected by quantitative coronary angiography (2.11±0.1 *vs* 1.89±0.09 mm, P<0.0001, q=34.67) but smaller than that detected by intravascular ultrasound (2.11±0.1 *vs* 2.19±0.11 mm, P<0.0001, q=12.61, Figure 2).

**Figure 3:**
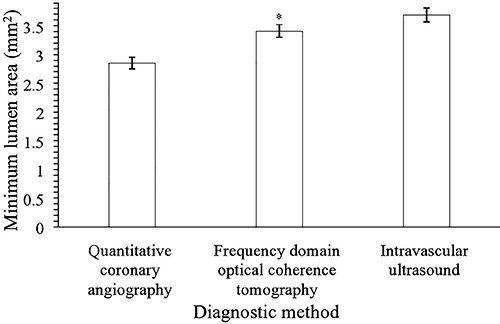
Minimum lumen area statistics. Data are reported as means±SD. Data of 250 patients were included in the analysis. *P<0.05 compared to the other diagnostic methods (ANOVA and Tukey *post hoc* test).

Minimum lumen area detected by FDOCT was larger than that detected by quantitative coronary angiography (3.41±0.01 *vs* 2.85±0.01 mm^2^, P<0.0001, q=80.274) but smaller than that detected by intravascular ultrasound (3.41±0.01 *vs* 3.69±0.01 mm, P<0.0001, q=40.137, Figure 3).

### Qualitative measurements

**Figure 4:**
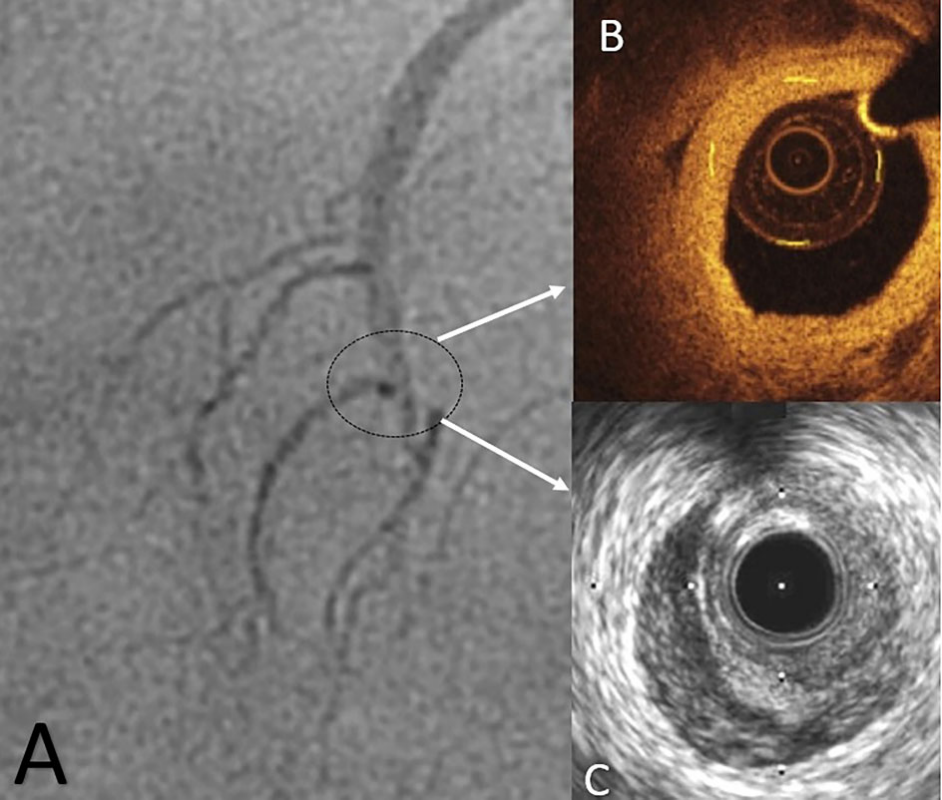
Pre-percutaneous coronary intervention images of a 52-year-old female. **A**, Clear coronary angiographic image. The black circle shows suspected coronary artery dissection. **B**, Frequency domain optical coherence tomographic image of the suspected part which clearly shows the lumen. Minimum lumen diameter: 2.08±0.09 mm, minimum lumen area: 3.25±0.01 mm^2^. **C**, Intravascular ultrasound image of the suspected part which clearly shows the lumen. Minimum lumen diameter: 2.18±0.1 mm, minimum lumen area: 3.48±0.01 mm^2^.

Quantitative coronary angiography showed diffuse lesions except for radiolucent flaps (Figure 4A). These lesions were clearly detected by FDOCT (Figure 4B) and intravascular ultrasound (Figure 4C).

FDOCT detected higher numbers of thrombus, tissue protrusion, dissection, and incomplete stent apposition than those detected by intravascular ultrasound (P<0.0001 for all, Table 3).

**Table 3:** Qualitative analyses of suboptimal lesion morphology.

Parameters	FDOCT	Intravascular ultrasound	P-values
Tissue protrusion	185 (74)	53 (21)	<0.0001
Incomplete stent apposition	71 (28)	33 (13)	<0.0001
Dissection	42 (17)	5 (2)	<0.0001
Thrombus	38 (15)	3 (1)	<0.0001

Data are reported as frequency (percentage) for 250 patients. FDOCT: Frequency domain optical coherence tomography. P<0.05 was considered significant (chi-squared test).

There was no significant difference between FDOCT, intravascular ultrasound, and quantitative coronary angiography for the site of the lumen.

Adverse effects, length of stay in the hospital, and permanent patient harm had not been reported regarding diagnostic procedures.

### Inter- and intra-rater agreement

Inter- and intra-rater agreement for quantitative coronary angiography (k=0.68), FDOCT (k=0.72), and intravascular ultrasound (k=0.71) were substantial.

### Complications of percutaneous coronary intervention procedure

Contrast-induced nephropathy was not reported for any patient. One case each of acute coronary occlusion, air embolism, slow flow, and coronary dissection was reported. Two cases each of thrombus formation and vasospasm were reported. Three cases of arrhythmia were reported and nine cases of difficulties in removing catheters were reported (Table 4).

**Table 4:** Complications related to percutaneous coronary intervention procedures.

Complications	Patients
Acute coronary occlusion (abrupt closure)	1 (0.4)
Air embolism (≥1 air bubbles)	1 (0.4)
Slow flow (slower than reported)	1 (0.4)
Coronary dissection (contrast agent found outside the coronary lumen)	1 (0.4)
Thrombus formation (haze found in projections)	2 (1)
Vasospasm (sudden vessel occlusion)	2 (1)
Arrhythmia (abnormal heart rhythm)	3 (1)
Difficulties in removing the catheter	9 (4)
Total	20 (8)

Data are reported as frequency (percentage) for n=250 patients.

## Discussion

FDOCT provided more accurate quantitative measurements than quantitative coronary angiography and intravascular ultrasound. The results of the current study were in line with the results of a multicenter prospective study (4), retrospective analyses (10–12), the phantom study (13), and a prospective study (14), but not in line with the OPINION trial (8) and the ILUMIEN III study (15). The reasons behind such discriminations of results are the gap between clinical trials and studies based on diagnostic performance in clinical practice (16). FDOCT visualizes the true lumen dimensions (4) because it provides cross-sectional images with a high spatial resolution (17), whereas intravascular ultrasound detects lumen dimension that is influenced by blood temperature, blood flow velocity, the incidence angle of the echo signal, and eccentric catheter placement (18).

In the study, six-to-eight projections of the left coronary arteries and two-to-three projections of the right coronary arteries were used for quantitative coronary angiography, while FDOCT and intravascular ultrasound did not require such projections for interpretations. In clinical practice, the use of FDOCT may allow significantly less angiographic acquisitions than intravascular ultrasound and quantitative coronary angiography.

FDOCT was more sensitive than intravascular ultrasound in detecting suboptimal lesion morphology. The results of the current study were in line with the results of the multicenter prospective study (4), retrospective analysis (11), and the ILUMIEN II study (19). FDOCT has superior visualization of the external elastic lamina through calcium without shadowing (20).

No adverse effect related to the diagnostic procedure and only a few complications related to percutaneous coronary intervention procedures were observed. The results of this study were consistent with the OPINION trial (8), retrospective analyses (21,22), and the ILUMIEN III study (15). The methods used in the study were safe.

As limitations of the study, tomography and ultrasound both require a guidewire for image acquisitions, and the lumen area can be minimally affected by the shadow of the guidewire (4). The effect of coronary pulsation on the area of the lumen was not evaluated. Tomography and ultrasound images were evaluated in the different phases of cardiac cycles. Intravascular ultrasound image resolution can be affected by frequency. Having no gold standard (e.g., histopathology or phantom study) and no diagnostic performance of data are major limitations of the study. For better evaluation of diagnostic methods, post-percutaneous coronary intervention images are necessary but the study did not report such results.

### Conclusions

FDOCT, intravascular ultrasound, and quantitative coronary angiography-guided percutaneous coronary intervention procedure are safe methods. More accurate and sensitive results of the coronary lumen may be possible to detect by FDOCT than coronary angiography and intravascular ultrasound. The FDOCT-guided percutaneous coronary interventions are recommended in patients with myocardial infarction. A prospective study is recommended to verify the results.
